# Identifying therapeutic targets for schizophrenia

**DOI:** 10.1038/s42003-021-02270-3

**Published:** 2021-06-10

**Authors:** Karli Montague-Cardoso

**Affiliations:** Communications Biology, https://www.nature.com/commsbio

## Abstract

There is a continual need to develop new therapies for neuropsychiatric disorders such as Schizophrenia, and identifying the underlying molecular processes remains challenging. Chadha et al. recently discovered a potential role for mTOR kinase activity disruption in Schizophrenia and further uncover the precise pathomechanism. Their study sheds further light on the role of mTOR in Schizophrenia and could inform the development of future therapeutic strategies for the condition.

Schizophrenia (SZ) is a neuropsychiatric condition that is experienced by 1% of the global population and requires life-long treatment. The need for a better understanding of the underlying biology of the condition and development of new treatments is ongoing. Numerous cellular signalling pathways have been implicated in SZ pathophysiology. One signalling cascade that has been shown to be disrupted in the SZ brain is mediated by the serine/threonine kinase mTOR. Under normal physiological conditions, mTOR signalling plays a crucial role in modulating neurotransmitter signals and mediating cell growth and survival. Recently, abnormalities in mTOR expression and phosphorylation have been observed in human SZ brains. The precise downstream effects of this however, have yet to be firmly established.

**Figure:**
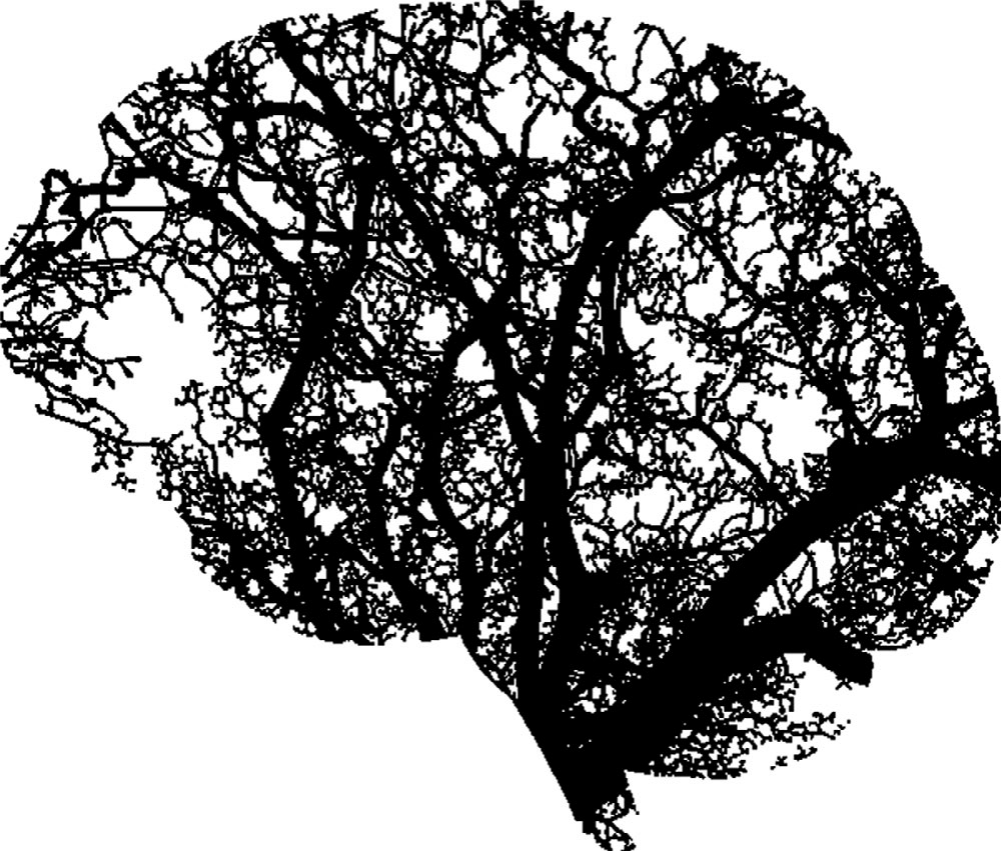
Pixabay

In a recent study published in *Molecular Psychiatry*, Chadha et al.^1^ used a combination of biochemical assays and kinome array profiling to understand the downstream effects of mTOR expression and activity disruption in postmortem SZ brains as well as brain tissue from rats treated with antipsychotics. In addition to the dysregulated mTOR kinase activity that they had previously identified in the postmortem SZ brains, they also saw reduced phosphorylation of several proteins (such as ribosomal protein S6 (S6RP)) that are downstream of mTOR complex I signalling and are known to play key roles in protein synthesis and cytoskeletal organization. Intriguingly, in the rat brain, following chronic treatment with the antipsychotic haloperidol, a therapy commonly used in SZ, they noted an increase in S6RP phosphorylation. This could therefore partially explain how haloperidol exerts its therapeutic effects. Chadha et al. went on to further investigate the role of mTOR kinase activity by inhibiting it with rapamycin in postmortem brain tissue, and compared the impact of this in SZ and control subjects using kinome arrays. They found that kinases in SZ brains were more effectively inhibited by rapamycin treatment. Analysis of the kinome array data also indicated that this sensitivity to rapamycin was likely due to AMP-activated protein kinase signalling.

Taken together, this study provides further understanding about the precise role of mTOR as a key regulator of changes in kinase activity in the SZ brain. This insight, in combination with the finding that haloperidol impacts downstream targets of mTOR, suggests that targeting mTOR signalling with novel or existing drugs could improve some symptoms of SZ, paving the way for the development of innovative treatments.
